# Workplace wellness programming in low-and middle-income countries: a qualitative study of corporate key informants in Mexico and India

**DOI:** 10.1186/s12992-018-0362-9

**Published:** 2018-05-09

**Authors:** Heather Wipfli, Kristin Dessie Zacharias, Nuvjote (Nivvy) Hundal, Luz Myriam Reynales Shigematsu, Deepika Bahl, Monika Arora, Shalini Bassi, Shubha Kumar

**Affiliations:** 1 Department of Preventive Medicine, Keck School of Medicine, University of Southern California, 2001 N. Soto Street, Los Angeles, CA 90089 USA; 2 Instituto Nacional de Salud Pública, Av. Universidad 655. Col. Santa María Ahuacatitlán, CP, 62100 Cuernavaca, Morelos Mexico; 3 Health Promotion Division, Public Health Foundation of India, Plot No. 47, Sector 44, Institutional Area, Gurgaon, 122002 India

**Keywords:** Workplaces, Wellness, Non-communicable diseases, Cancer prevention, Mexico, India

## Abstract

**Background:**

A qualitative study of key informant semi-structured interviews were conducted between March and July 2016 in Mexico and India to achieve the following aims: to explore corporations’ and stakeholders’ views, attitudes and expectations in relation to health, wellness and cancer prevention in two middle-income countries, and to determine options for health professions to advance their approach to workplace wellness programming globally, including identifying return-on-investment incentives for corporations to implement wellness programming.

**Results:**

There is an unmet demand for workplace wellness resources that can be used by corporations in an international context. Corporations in India and Mexico are already implementing a range of health-related wellness programs, most often focused on disease prevention and management. A number of companies indicated interest is collecting return on investment data but lacked the knowledge and tools to carry out return-on-investment analyses. There was widespread interest in partnership with international non-governmental organizations (public health organizations) and a strong desire for follow-up among corporations interviewed, particularly in Mexico.

**Conclusions:**

As low-and middle-income countries continue to undergo economic transitions, the workforce and disease burden continue to evolve as well. Evidence suggests a there is a growing need for workplace wellness initiatives in low-and middle-income countries. Results from this study suggest that while corporations in India and Mexico are implementing wellness programming in some capacity, there are three areas where corporations could greatly benefit from assistance in improving wellness programming in the workplace: 1) innovative toolkits for workplace wellness initiatives and technical support for adaptation, 2) assistance with building partnerships to help implement wellness initiatives and build capacity, and 3) tools and training to collect data for surveillance as well as monitoring and evaluation of wellness programs.

## Background

Major lifestyle changes around the world driven by globalization and rapid urbanization are resulting in the global rise of non-communicable diseases (NCDs) [1]. The World Health Organization (WHO) estimates 40 million deaths each year are attributed to NCDs. More than 80% of these deaths occur in low and middle-income countries (LMICs) with cardiovascular diseases, cancers, respiratory diseases, and diabetes being the leading causes of death. Physical inactivity, poor diet, as well as tobacco and alcohol use are the leading modifiable behavioral risk factors in the development of obesity, hypertension, and high levels of glucose and fat in the blood, which are the leading causes of NCDs worldwide [2]. Increased access to and consumption of cheap processed foods, the creation of sedentary job markets, and increased access and availability to tobacco and alcohol products have all helped to drive global risk factors for NCDs. The NCD epidemic not only adversely affects quality of life, but also inhibits economic prosperity from the individual to national level [3].

Growing concern regarding the rise in NCDs has resulted in a number of political actions at the international level. In 2011, the United Nations General Assembly (UNGA) adopted a Political Declaration on NCDs, which acknowledged the global burden, threat to economics, and need for action from multi-sectoral leaders on a local, national, and international level [4]. In follow up to the UN Declaration, WHO has adopted a number of NCD-related resolutions including the global target to reduce NCD deaths by 25% by 2025 and the WHO Global NCD Action Plan [5]. In 2015, the UNGA also adopted the Sustainable Development Goals (SDGs), which recognized the negative impact of NCDs in promoting and achieving sustainable social, economic and environmental development [6].

Repeatedly within these political documents, workplaces are identified as target areas for NCD prevention and control. In high, middle, and low- income countries alike, wellness programming within the workplace has been identified as a strategic method to prevent and treat NCDs as more than half of the global population spends one-third of their adult lives at work [7]. The economic benefit of scaling ‘best buy’ interventions such as smoke-free workplaces in LMICs alone is estimated as high as $377 billion [3].

Engagement of workplaces in NCD prevention does not just benefit public health. Over time chronic NCDs can become expensive for companies to treat and lead to a decrease in productivity. Increasingly, corporations have realized there is a potential return on investment (ROI) in successful workplace wellness programming [3]. Due to the correlation between a healthy workforce and strong finical performance, workforce health metrics can be increasingly seen as a measure of corporate success [8]. In in LMICs however, the WHO and the World Economic Forum (WEF) estimates between 2011 and 2025 a loss of more than $7 trillion due to NDCs [9]. Despite this staggering number, only 33% of business leaders indicated serious concerns about NCDs in 2013 [10].

Globally only 29% of companies have implemented wellness programs [11]. In the United States (U.S.) this percentage grows to 51% for employers with at least 50 employees, with larger companies offering more sophisticated programming [12, 13]. In a global workplace survey, 49% of companies implementing wellness programs reported lower health care costs after one year and 80% of companies indicated improvements after five years. In addition, employers also saw reductions in absenteeism due to illness, presenteeism, and attrition [11]. In the United States (U.S.), employees who participate in wellness initiatives are more likely to smoke less, exercise more, and better manage their weight. In addition, participation over time leads to reduced health care costs, absenteeism as well as increased productivity [12, 13].

Wellness programming can take many forms including medical screening, disease prevention (primary and primordial prevention), and disease management interventions. Programming can come from a variety of sources including an outside vendor specializing in programming, health insurance plans, governmental resources or in-house company design [12]. In the U.S., non-profits and public health organizations, including the American Cancer Society (ACS) and the American Heart Association (AHA), have engaged directly with corporations in workplace wellness initiates. These organizations have developed tools for tobacco cessation, nutrition, and physical activity, tailored to the U.S. population for little or no cost. Realizing the success of these programs in the U.S., transnational companies have increasingly sought assistance to expand the wellness programs to their international sites. This, however, has been challenging as domestic non-profit organizations often lack the capacity and experience needed to adapt the existing resources in culturally appropriate and politically relevant ways in diverse LMICs where the companies have workers.

This paper presents the results of a research project carried by the University of Southern California (USC) which aimed to collect data to support U.S. non-profits, specifically ACS that provided core funding, to deciding if and how to expand technical assistance in the area of workplace wellness to include workers based in LMICs. While several larger scale quantitative surveys have been implemented globally to assess the wellness programming landscape [10, 11], there is less qualitative data informing the specifics of on the ground practices, implementation challenges and overall views, attitudes and expectations in relation to health in specific communities. This paper seeks to address that gap by providing insight from a semi-structured interview design that allowed for the collection of such valuable insights as well as engaged corporations in a dialogue where cultural norms and local customs could be uniquely addressed and acknowledged during data collection.

The study focused on corporations’ and stakeholders’ views, including attitudes and expectations in relation to workplace health and wellness to better understand the wellness landscape in two LMICs - India and Mexico. India and Mexico were targeted due to the fact that many U.S. based transnationals have international offices in these two countries and seek culturally relevant and targeted interventions for these locations, as well as the fact that both countries suffer from large NCD burdens. In 2016, for example, the top six causes death in Mexico were NCDs including cardiovascular disease, diabetes, cirrhosis and chronic respiratory diseases [14]. The disease burden in India is also heavily due to NCDs with cardiovascular diseases as the top cause of death in the country. Chronic respiratory disease and diabetes rank among the top five causes of death as well [15]. Results of the study suggest concrete next steps needed to enhance workplace health and wellness programing in LMICs.

## Methods

A qualitative study was conducted through international key informant semi-structured interviews in India and Mexico between March and July 2016 to achieve the following aims: to explore corporations’ and stakeholders’ views, attitudes and expectations in relation to health, wellness and cancer prevention, and to determine options for health organizations to be more responsive, strategic and innovative in their approach to corporations and health and wellness programming globally, including identifying ROI incentives for corporations to implement wellness programming.

Twenty semi-structured key informant interviews were included in the following analysis: 10 corporate stakeholders in India, and 10 corporate stakeholders in Mexico. Key informants in India and Mexico represented a range of industries, company size and included both national and transnational corporations (Table 1 and Table 2). The sample size was determined by feasibility and intentionally represented the diversity of the workplace landscape in each country by size and sector in an effort to 1) obtain preliminary qualitative data of the interworkings of wellness programming challenges to provide context for and complement existing quantitative survey data regarding workplace wellness, 2) identify corporate leaders in each sector as potential partners for wellness programming implementation research in future.

**Table 1:** Overview of stakeholders in Mexico

City/Region	Industry	Workforce Size	National/Multinational
Hidalgo	Manufacturing	260	National
Mexico City	Financial services	1800	National
EDOMEX	Distribution	5528	National
Mexico City	Government Tourism	700	National
Mexico City	Government Health Research	1600	National
Puebla	Textiles	800	National
Puebla	Education	1660	National
Puebla	Telecommunications	1300	National
Cuernavaca	Manufacturing	90	National
Cuernavaca	Manufacturing	4800	Multinational

**Table 2:** Overview of stakeholders in India

City/Region	Industry	Workforce Size	National/Multinational
Delhi	Medical	100,000	National
Chennai	Technology	353,000	Multinational
Bangalore	Technology	240	Multinational
Bangalore	Technology	60	National
Bangalore	Technology	15,000	Multinational
Bangalore	Technology	150	National
Delhi	Government Agency	1300	National
Bangalore	Manufacturing	67,000	Multinational
Chennai	Manufacturing	170,000	Multinational
Delhi	Manufacturing	13,000	Multinational

International interview guides were developed in conjunction with in-country partners at Instituto Nacional de Salud Publica (INSP) in Mexico and Public Health Foundation of India (PHFI). First, key translational themes of interest were identified and used to guide the core questions to be asked of all respondents. Then questions and interview procedures were drafted and shared with the entire team for comments and revisions based on feasibility and applicability. The final guide was distributed to all team members and hard copies were taken to each interview and followed to ensure consistency. The study was approved by the Internal Review Board at the USC and Institutional ethics committees in India (Referen*ce No. TRC-IEC-298/16*) and Mexico. In-country partners facilitated the identification and recruitment of corporate stakeholders. In India, these corporations were purposively selected from three cities (Delhi, Bangalore and Chennai) in an effort for geographic representation as well as representation from three large corporate centers.

Semi-structured individual interviews were conducted in-person with corporations in India and Mexico in conjunction with site visits by the research team. Consent was obtained from each key informant prior to the start of the interview. Questions examined why corporations do or do not have workplace wellness programs, and if they have them, then the goals and objectives of their corporate wellness programs, and the departments or areas in which wellness programs are based in the corporation. Questions also examined challenges and barriers corporations face when investing in wellness programs, and how corporations have or have not developed a culture of health within their organizations.

All interviews in India were conducted in English. Most interviews in Mexico were conducted in English and those conducted in Spanish were administered by a bilingual member of the USC study team, and translated real-time to English by a professional translator. Most interviews were digitally recorded and professionally transcribed. A few international partners preferred interviews not be digitally recorded, but analysis based on detailed written records were included in the following analysis. A portion of the transcripts were verified against the recordings for quality control. Transcribed interviews were coded using Dedoose, a web-based qualitative data analysis software [16]. A codebook was developed for the interview guide to standardize the coding process. A preliminary codebook was created based on each interview guide and revised to include any relevant missing codes based on interviewee responses. After all interview transcripts were coded, a list of salient themes emerged as the most common and prominent issues identified by the respondents based on the identified codes. Codes were evaluated using summary statistics.

## Results

Key informants indicated corporations in India and Mexico are already implementing a range of health-related wellness programs, most often focused on disease management rather than overall health promotion or reducing modifiable risk factors for NCDs. Findings in Mexico and India were similar in regards to wellness programming focus areas and approaches (Table 3).

**Table 3:** Health programming initiatives in Mexico and India

Health Area/Topic Focus	Mexico	India
Tobacco Control/Cessation	50% of respondents indicated their workplaces were smoke free	70% of respondents indicated their workplaces were smoke free although they reported significant compliance challenges.
Physical Activity	80% of corporations indicated some type of physical activity program within the workplace.	70% of corporations indicated some type of physical activity program within the workplace including exercise facilities or classes offered at the worksite.
Nutrition	50% of corporations surveyed stated there was a nutritionist onsite who designed healthy meals for the cafeteria.	50% of corporations surveyed identified wellness programming related to nutrition and healthy diet.
Disease Detection and Management	70% of corporations indicated a vaccination campaign 50% corporations indicated NCD prevention efforts and diagnostic screenings	Only 20% of corporations indicated vaccination campaigns or programs. 80% of corporations indicated diagnostic screenings for NCDs usually through annual checkups.

### Mexico

#### Health culture

Corporate health culture in Mexico is largely defined by occupational health and labor legislation which has led to a large intersection between medical care, the government, and the workplace. Most notably, the Mexican Institute for Social Insurance known in Spanish as “Instituto Mexicano del Seguro Social” or “IMSS” is responsible for providing health insurance to all non-governmental salaried workers and their families in Mexico. The program is compulsory and funded through employee, employer, and government contributions [17]. In addition to providing medical coverage, IMSS delivers preventive care programs through the “PREVENIMSS” initiative within the workplace [18]. PREVENIMSS provides twice annual health campaigns in the workplace with services including vaccinations, health talks or trainings, and medical examinations for employees with a focus on NCD prevention. Corporations that were more aware of the resources available at PREVENIMSS were able to request and receive more services. Most corporations indicated that they also receive support from private business including pharmaceutical, healthcare, food, and beverage companies in addition to PREVENIMSS campaigns. Some companies noted that the introduction of PREVNIMSS had resulted in less company engagement in health promotion as it was now no longer seen as their responsibility, but instead something that the government would provide to their employees.

#### Priority health risks

Respondents indicated obesity, hypertension, diabetes, cancer, and orthopedic ailments as the major priority health risks among employees. Tobacco use was acknowledged as a major health risk as well. Corporations across sectors estimated tobacco use to be as high as 20–30% of employees, in some cases.

#### Programming and communication

80% of corporations indicated some type of physical activity program within the workplace. Some corporations offer exercise classes on site including dance, yoga, and cross training or created company soccer, basketball, or volleyball teams and tournaments as well as walks and runs. A few corporations initiated fitness challenges which included body mass index (BMI) measurement and weight loss tracking. Half of corporations surveyed stated there was a nutritionist onsite who designed healthy meals for the cafeteria. Two companies noted specialized weight loss programs with nutritional support for employees. Multiple corporates mentioned employee resistance to these programs and their preference to traditional Mexican cuisine. Wellness programming communication tactics varied by job type. For jobs that were primarily desk-based, internet communications such as emails were used to communicate wellness programming, while environments where jobs were more labor intensive, utilized posters, physical message boards, and in-person messaging from unit supervisors. Interviewees indicated the most useful tools to be low-cost or free health education materials or talks. Most corporations reported turning to an external organization for some kind of support. External support was largely in the form of guest speakers, who would provide a health talk in their specialized field or expertise. The speakers nearly always came from nearby hospitals and focused on disease management.

#### Motivations

In Mexico, the rationale for investment in wellness programming stemmed from three areas: ROI, corporate social responsibility (CSR), and recruitment and retention strategy. Corporations that do calculate ROI highlighted the decrease in absenteeism and major medical expense for costly illnesses such as cancer. One corporation explained they use ROI data to justify wellness programming budgets to senior management. A number of interviewees indicated interest in collecting ROI data but lacked the knowhow and tools to carry out ROI analyses. Beyond meeting all occupational health legislative requirements, some corporations emphasized the importance of CSR in regards to workplace wellness. One company explained *“It’s mainly because the company is socially responsible. … it’s not just about being in compliance …, it’s more about the company wants to give its employees the ideal conditions for them to do their work, which is also good for the company, and socially, and for health as well.”*

#### External stakeholders and partners

All corporations surveyed received support from the federal government for health and wellness programming. The majority of corporations receive program implementation support from IMSS for twice annual PREVENIMSS campaigns. Most corporations also receive support from wellness initiatives through external partnerships with pharmaceutical, healthcare, food, and beverage companies. Often this support was given in exchange for professional services. The majority of corporations were interested in receiving support from external public health organizations.

#### Challenges

The most commonly cited challenges among corporations in instituting wellness programming were raising overall awareness about health and disease prevention and actually changing and sustaining positive health behaviors. Corporations cited low education and health literacy levels as key contributors to these challenges. One interviewee explained the challenge of sustained behavior change in describing tobacco control efforts *“One of …the concerns that we have regarding the measures that we’re taking, is not to displace a problem, meaning that, we would not like employees to just not smoke inside the company, but rather raise awareness and achieve, in the long run, that they do not smoke at all.”* Another respondent explained the difficulty of communicating the importance of prevention rather than treatment to employees. They explained *“You don’t have to be sick to go find help. You want to avoid... prevent…these types of situations.”* Another area of concern identified was program cost and limited staff resources. In addition, cultural norms and traditional diets were seen as a challenge to the success of nutrition and diet programming, as employees were less willing to adopt new meal options.

### India

#### Health culture

Health culture in India was not consistently defined as in Mexico and varied based on whether the company was a national or transnational company. The approaches to company’s health culture ranged from holistic wellness to minimal engagement. The transnational corporations tended to have structured wellness programming adapted to the Indian context as compared to national corporations, which were aware of the health issues in their population but did not have the resources to develop strategic programming. The majority of the corporates identified health insurance or benefits for their employees and family members as part of companies’ wellness programming. Similar to findings in Mexico, the government played a role in how corporations viewed their wellness programming responsibilities. For example, smoking was less of a concern for some corporations as there was a perceived decrease in smoking rates and perceived lack of need for such programs due to Indian tobacco control law, which prohibits smoking in public places. Almost all workplaces had smoke-free policies, with varying degrees of enforcement. Corporations also felt that government funded tobacco cessation programs were available so they did not need to invest in those services.

#### Priority health risks

In India, priority health risks included obesity, largely attributed to lifestyle factors, such as diet, exercise, and tobacco use. In addition, many corporations also indicated that mental health issues were of concern, particularly in terms of stress management. Physical activity was often identified as an avenue for corporations to address health issues in their workforce with 70% of corporations indicating providing some form of physical activity programming such as runs, marathons, yoga and meditation classes. In addition, 70% of respondents indicated workplaces were smoke free and few had tobacco cessation programming. Many corporations indicated minimal programming related to cancer screening, however some did express interest in integrating cancer-related programming.

#### Programming and communication

Among some corporations, health talks or presentations were mentioned as an important intervention to improve employee health. Many corporations mentioned having physical activity programs including corporate runs marathons, yoga and meditation classes. Several corporations discussed office policies as part of their employee wellness initiatives. These policies generally focused on diet and nutrition. One corporation introduced a “wellness menu” which included more fruit and vegetables as well as meals that are low in sugar and salt. In addition to in-person talks, emails, notice boards, mobile platforms, such as *Whatsapp*, SMS, and social media were viewed as effective modes of communication and program delivery for several corporations, regardless of industry or company size.

#### Motivations

In India, ROI was the most common rationale for wellness programs among transnational corporations, while national corporations did not have in place robust mechanisms to measure ROI and were more reluctant to frame health in terms of financial return. National corporations often emphasized the need for corporations to contribute to India’s development and efforts to improve the health of all its citizens whereas transnational corporations were much more comfortable discussing the quantitative measures they use when deciding between programs and evaluating their effectiveness.

#### External stakeholders and partners

Most corporates reported turning to an external organization for some kind of support. External support was largely in the form of guest speakers, for providing a health talk in their specialized field or expertise from local health care organizations, such as hospitals. While several corporates indicated external organizational partnerships, only one stated they had implemented a tool or program provided by an external partner.

#### Challenges

Particularly for voluntary programs, employee participation was seen as a challenge, including both initial participation as well as continued engagement. Notably, there was an apparent competing interest in some corporations as they viewed high levels of participation in wellness programming as a barrier to productivity: *“…we don’t like our employees to just concentrate [on] yoga...) they come here to work.”* While smoke free policies were in place, corporations did not often have cessation programming on site. Some key informants stated enforcement of smoke-free policies as difficult to implement.

## Discussion

Country specific conditions greatly impact the results found in this study. Political and legislative polices such as the government run “PREVNIMSS” campaign entirely shapes and standardizes the workplace wellness culture in Mexico. Such a structure is not in place in India resulting in more varied implementation strategies. Epidemiologically, similar NCDs are leading causes of death in both countries due to similar modifiable risk factors associated with lifestyle. As a result, priority health risks were similar in both countries.

Results from this study suggest that while corporations in India and Mexico are implementing wellness programming in some capacity, there are three areas where corporations could greatly benefit from assistance in improving wellness programming in the workplace: 1) innovative toolkits for workplace wellness initiatives and technical support for adaptation, 2) assistance with building partnerships to help implement wellness initiatives and build capacity, and 3) tools and training to collect data for surveillance as well as monitoring and evaluation of wellness programs.

### Wellness tools

Evidence-based toolkits and educational resources that can easily be adapted are ideal resources for corporations to implement wellness programming. Cultural adaptation and tailoring are essential in developing effective workplace wellness programming. In Mexico one respondent explained, “…When you talk about Latin American, well we have a very wide spectrum.”, highlighting the need for a shift away from a “one-size-fits-all” approach to programming that takes into account cultural norms and beliefs of the population. Even in regards to language, key informants indicated translation of materials from English to Spanish is not sufficient as country-specific dialects and terminology must be taken into account. In addition, tools must be designed with country-specific legislation in mind. Similar needs for adaptation exist in India where cultures and languages vary throughout the 29 states and where the workforce diversity ranges from agriculture to service industry to the growing information technology and software industries [19]. Occupational labor regulations, tobacco control policies, and access to medical care all stem largely from domestic legislation and as a result dictate the parameters of workplace wellness tools.

### Building partnerships

Leveraging existing local resources to implement wellness programming and build capacity would help to ease the financial burden of effective wellness programming. In addition, partnerships lend the expertise needed. In both countries there were corporations that had in place model wellness programs that were culturally and political appropriate, and transnationals, in particular those with in-depth knowledge, indicated their willingness and interest in sharing their expertise with others in the country. Developing a local network of corporations, medical centers, public health organizations, and universities can create a forum for resource sharing as well as implementation support tailored to the specific cultural norms of that community. In the case of Mexico in particular, the existing PREVENIMSS infrastructure can be enhanced by these partnerships to improve the quality and effectiveness of programming.

### Surveillance / monitoring and evaluation

Despite perceived value of wellness initiatives, few corporations were calculating actual ROI. Some corporations were collecting health data, but few were able to use the data effectively to inform wellness initiatives. Programs could be improved by collecting additional specific indicators more relevant to wellness and prevention. Health surveillance data are needed to inform evidence-based wellness initiatives, and, monitoring and evaluating wellness programming is needed to develop evidence-based programs for LMIC settings. Corporations are in need of training and simple solutions to collect, manage, and analyze data as well calculate ROI. In addition, data collection must be conducted in ways that respect the privacy and confidentiality of individuals. The collection of sensitive personal data by employers may allow for individualized care, but it also raises ethical challenges that must be considered by corporations as they globalize their practices.

### Limitations of the study

While the study aimed to gather insights from a diverse sample of corporations across a variety of sectors in two different countries, recruitment of corporations into the study was primarily based on in-country partners’ network with corporations and should be taken into consideration in regards to generalizability of the results.

The small and diverse study sample provides insightful, suggestive data of a cross-section of the workplace wellness landscape in India and Mexico, not necessarily representative of one sector or corporation size across either country. Further research is needed to focus on specific facets of the workplace landscape to reach an appropriate saturation point for improved generalizability.

Notably, both Mexico and India have a large informal workforce, which would not be addressed through corporate workplace wellness programs. In addition, although the participants in Mexico varied in regards to job type, those interviewed in India were predominantly professional rather than manual laborers. Therefore, there are limitations in the generalizability across industries in India. Regional differences across Mexico and India are also a limitation in the generalizability of this study. Further research to include a wider range of industries and regions in both countries, as well as countries in other regions is recommended. These results do no claim to be representative of the vast workplace landscape across Mexico and India, but rather informative data from sentinel corporations that could serve as potential partners for workplace wellness program implementation in the future.

Despite collaborative creation of interview guides with international partners for cultural appropriateness and relevance, the interview guides did not undergo rigorous pre-testing or translation or back-translation into Spanish prior to interviews introducing potential bias. Additional pilot testing and a rigorous translation protocol are needed in further research efforts to improve reliability.

## Conclusions

Corporations in India and Mexico are already implementing a range of health-related programs, most often focused on disease prevention and management. Corporate health culture is most greatly influenced by government regulation, transnational ties, and country specific customs. Large multi-nationals tended to have a workplace wellness framework instituted on a corporate level. Without such guidance, smaller corporations tended to have a less structured wellness program in place. In all cases, involvement and modeling of healthy behavior by company leaders was key to the corporation’s health culture and implementation of wellness programming. Corporations could greatly benefit from assistance in improving wellness programming in the workplace through innovative toolkits for workplace wellness initiatives and technical support for adaptation, assistance with building partnerships to help implement wellness initiatives and build capacity, and tools and training to collect data for surveillance as well as monitoring and evaluation of wellness programs.

Corporations in India and Mexico indicated interest to partner with external groups, including public health organizations. Similar to what corporations have done in the U.S., partnerships with public health organizations may fill the gaps between what the government and workplace can provide in LMIC settings. Further research is warranted to assess the effectiveness of adaptation of best practices in workplace wellness initiatives and what works to motivate corporate investment in wellness programming.
